# **Socioeconomic development index (SDI) gradients and high BMI-Driven pan-cancer burden: a global burden of disease study on mortality**,** disability**,** and health inequities (2015–2021)**

**DOI:** 10.1186/s12889-025-24531-0

**Published:** 2025-10-02

**Authors:** Zhuang Ma, Sixuan Zou, Ruoxuan Liu, Song Li, Zhenjiang Li

**Affiliations:** 1 Neurosurgery, Huaihe Hospital of Henan University, Kaifeng, 475000 China; 2 Medical department, Huaihe Hospital of Henan University, Kaifeng, 475000 China; 3 Surgical Department, Huaihe Hospital of Henan University, Kaifeng, 475000 China

**Keywords:** High BMI, Pan-Cancer burden, Socioeconomic development index (SDI), Global burden of disease (GBD), Health disparities, Disability-Adjusted life years (DALYs)

## Abstract

**Background:**

The rising prevalence of high body mass index (BMI) has become a critical driver of global oncologic morbidity and mortality, yet its pan-cancer burden remains poorly characterized across socioeconomic development strata. This study investigates the geographic, temporal, and sex-specific disparities in high BMI-attributable cancer burden, stratified by the Socioeconomic Development Index (SDI), to inform precision public health strategies.

**Methods:**

Leveraging the 2021 Global Burden of Disease (GBD) dataset, we analyzed age-standardized mortality, disability-adjusted life years (DALYs), and years of life lost (YLLs) for 17–23 countries across Asia and globally. SDI-stratified analyses evaluated temporal trends (2015–2021) and cancer-type contributions, while multivariable models assessed associations between income inequality (Gini coefficient), healthcare capacity, and metabolic risk exposure.

**Results:**

Marked disparities emerged across SDI gradients: high-SDI nations exhibited 6.7-fold higher mortality rates (e.g., Malaysia: 4.40 vs. Bangladesh: 0.65/100,000) and concentrated burdens in colorectal (40.5% DALYs) and breast cancers (27.0% DALYs), contrasting with distributed burdens in low-SDI regions (no cancer > 15.6% DALYs). Gender disparities highlighted male predominance in liver (+ 8.4 DALY difference) and colorectal cancers (+ 5.1), while female-specific malignancies (e.g., uterine cancer) retained consistent burdens across SDI levels. Temporal analyses revealed accelerated DALY reductions in middle-SDI regions (-4.5% annual percent change [APC]) but rising breast cancer burdens in low-SDI settings (+ 1.2% APC). Economic inequality (Gini > 0.40) correlated with elevated mortality (Turkey: 123.1/100,000), independent of GDP, underscoring synergistic impacts of BMI and sociodemographic inequities.

**Conclusion:**

High BMI-driven pan-cancer burden is profoundly shaped by SDI gradients, reflecting interactions between metabolic risk, healthcare access, and socioeconomic determinants. Tailored interventions—prioritizing colorectal and breast cancers in high-SDI regions and addressing systemic inequities in low-SDI settings—are critical to mitigating the dual burden of obesity and cancer in transitioning populations.

## Introduction

The global burden of cancer remains a pressing public health challenge, with an estimated 19.3 million new cases and 10.0 million deaths reported in 2020 alone [1]. While traditional risk factors such as tobacco use and infections dominate oncologic epidemiology, emerging evidence underscores the escalating role of metabolic disorders—particularly high body mass index (BMI)—as a critical driver of cancer incidence and mortality [2]. Recent Global Burden of Disease (GBD) studies estimate that high BMI contributed to 4.5% (95% UI: 3.4–5.6) of cancer-related deaths globally in 2019, with pronounced heterogeneity across cancer types and regions [3]. However, existing analyses have predominantly focused on individual malignancies (e.g., colorectal or breast cancers), leaving the pan-cancer burden attributable to high BMI—and its interaction with socioeconomic development—poorly characterized.

Socioeconomic Development Index (SDI), a composite metric integrating income, education, and fertility rates, has emerged as a key determinant of health disparities in non-communicable diseases [4]. While high-SDI nations exhibit greater exposure to obesogenic environments (e.g., sedentary lifestyles, processed food consumption) [5], low-SDI regions face dual burdens of rising obesity prevalence and limited healthcare capacity to manage advanced malignancies [6]. This paradox is exemplified by Southeast Asia, where economic growth has driven a 35% increase in age-standardized BMI since 1990, yet cancer screening coverage remains below 20% in countries like Malaysia and Thailand [7]. Such disparities suggest that SDI gradients may modulate both the risk exposure (high BMI) and health system response (early detection, treatment), creating complex patterns of obesity-driven cancer burden.

Current limitations in understanding this relationship are threefold. First, prior studies have inadequately addressed sex-specific disparities in high BMI-related cancers. For instance, while endometrial cancer shows strong female predominance, emerging data suggest that males with high BMI face elevated risks for aggressive subtypes of prostate and liver cancers [8]. Second, the temporal evolution of this burden remains unclear: although high-income countries report declining obesity-related gastric cancer mortality [9], parallel trends in transitioning economies (e.g., India, Nigeria) are masked by competing risks from infections and undernutrition [10]. Third, no study has systematically evaluated how income inequality (measured by Gini coefficient) interacts with SDI to amplify or mitigate geographic disparities in cancer outcomes—a critical gap given that countries with similar SDI scores (e.g., Malaysia vs. South Korea) exhibit 2.1-fold differences in obesity-related DALYs [11].

To address these gaps, we leveraged the 2021 GBD dataset to conduct a comprehensive analysis of high BMI-driven pan-cancer burden across 21 countries, stratified by SDI quintiles. Our study aims to: (1) Quantify 2015–2021 temporal trends and geographic disparities in age-standardized mortality, DALYs, and YLLs for high BMI-attributable cancers across SDI quintiles in 21 Asian and global countries; (2) Identify SDI-stratified cancer-type-specific burden distributions (e.g., colorectal, breast cancer) and their association with metabolic risk profiles; (3) Evaluate the independent and interactive effects of income inequality (Gini coefficient), healthcare capacity, and sex-specific vulnerabilities on high BMI-driven cancer outcomes. This research provides novel insights into the syndemic interaction between obesity and cancer, offering evidence to guide SDI-tailored prevention policies and mitigate global health inequities.

## Materials and methods

### Geospatial analysis of high BMI-related cancer burden

The study analyzed age-standardized metrics from the Global Burden of Disease (GBD) 2021 database to assess the pan-cancer burden attributable to high body mass index (BMI) across 17 Asian countries. Mortality, disability-adjusted life years (DALYs), and years of life lost (YLLs) were extracted using the GBD Compare Tool (Institute for Health Metrics and Evaluation [IHME]), with geospatial alignment ensured by mapping country-specific ISO 3166-1 alpha-3 codes. Territories with unresolved geopolitical status (*n* = 3) were excluded to maintain analytical consistency. Spatial distributions of mortality and DALY rates (per 100,000 population) were visualized through Google GeoChart API (v1.0) and stratified by Sociodemographic Index (SDI) quintiles. Spatial clustering was assessed using Moran’s I statistic (coefficient range: 0.21–0.38, *p* < 0.001), confirming significant global autocorrelation in high BMI-driven cancer burden. Data normalization applied the WHO 2000–2025 standard population. Sensitivity analyses evaluated spatial clustering robustness using Moran’s I and local Getis-Ord Gi statistics (z-scores > 2.58, *p* < 0.05 for hotspot identification). Ethical approval was waived per institutional guidelines as all data were fully anonymized and publicly accessible under GBD data use agreements.

### SDI-stratified comparative risk assessment

A comparative analysis of BMI-attributable cancer burden was conducted across 22 countries using GBD 2021 estimates standardized to the WHO World Standard Population. Twelve malignancies with IARC Group 1 evidence for obesity association (e.g., colorectal, postmenopausal breast, and endometrial cancers) were selected based on meta-analyses by Lauby-Secretan et al. (2016). Age-standardized mortality and DALY rates were calculated through compartmental modeling integrating:

1. Population-attributable fractions (PAFs) derived from comparative risk assessment;
2. Theoretical minimum risk exposure distribution (BMI 21–23 kg/m²);
3. Exposure-response relationships from pooled cohort studies.

SDI stratification followed GBD 2021 protocols (low: 0–0.35; middle: 0.36–0.69; high: ≥0.70). Uncertainty intervals (UIs) were generated through 10,000-cycle Monte Carlo simulations accounting for exposure variance, relative risk heterogeneity, and PAF estimation error. Statistical significance thresholds were adjusted for multiple testing using Benjamini-Hochberg false discovery rate correction (FDR < 0.05).

### Sex-specific disparity analysis

Sex-stratified DALY rates for eight high BMI-associated cancers (gallbladder, ovarian, renal, pancreatic, liver, colorectal, breast, and uterine cancers) were extracted from GBD 2021 Asian datasets. Data were filtered to retain only age-standardized rates (metric_name="rate”; age_name="age-standardized”). Male-to-female disparity (Δ) was calculated as:

$$\mathrm\Delta=\mathrm{Male}\;\mathrm{DALY}\;\mathrm{rate}-\mathrm{Female}\;\mathrm{DALY}\;\mathrm{rate}$$

#### 95% confidence intervals (CIs) were computed via error propagation

$$\begin{aligned}\mathrm{CI}=\lbrack&\mathrm\Delta-({\mathrm{male}}_{\mathrm{lower}}-{\mathrm{female}}_{\mathrm{upper}}),\\&\mathrm\Delta+({\mathrm{male}}_{\mathrm{upper}}-{\mathrm{female}}_{\mathrm{lower}})\rbrack \end{aligned}$$

Statistical significance was defined as CI exclusion of zero. Disparities were visualized through bidirectional waterfall plots (Plotly v5.18.0) with male-excess (blue) and female-excess (red) bars. Sensitivity analyses confirmed stability across alternative CI estimation methods.

### Socioeconomic contextual analysis

The analytical framework utilized nationally representative data from the Global Burden of Disease Study 2024 (IHME) and the World Bank Poverty and Inequality Platform (2024) covering 23 Asian countries. Cancer mortality rates (deaths per 100,000 population) were paired with socioeconomic indicators, including Gini coefficients (income inequality) and GDP per capita (adjusted for purchasing power parity), to assess contextual associations. Data integrity was ensured through rigorous validation against source repositories, with exclusion criteria applied to incomplete or non-harmonized entries. Bivariate analyses were conducted using Pearson’s correlation coefficients to quantify relationships between mortality rates and socioeconomic variables, supplemented by linear regression models to adjust for population size heterogeneity. Interactive scatterplots were generated programmatically via Plotly.js (v2.18.0) to visualize non-linear trends, with axes scaled to standardized ranges (Gini: 0.25–0.45; GDP: PPP-adjusted USD) to enhance cross-country comparability. All analyses were stratified to account for region-specific confounders, including prevalence of high BMI-driven cancer DALYs, with sensitivity tests confirming robustness to outlier exclusion. Statistical significance was evaluated at α = 0.05 using two-tailed tests, and analytical workflows were implemented in R (v4.3.1) with full reproducibility protocols.

To address potential confounding, multivariable linear regression models were employed, adjusting for population size, healthcare access and quality index (HAQ), and urbanization rate. Confounding by GDP per capita and income inequality (Gini coefficient) was explicitly controlled via fixed-effects modeling. The GBD framework inherently accounts for demographic confounding through age-standardization to the WHO World Standard Population, and comparative risk assessment methods isolate the attributable burden of high BMI by referencing a theoretical minimum risk exposure distribution (BMI 21–23 kg/m²). Sensitivity analyses excluded outliers and verified results using robust regression techniques, with all models validated via 1,000-cycle bootstrap resampling to assess coefficient stability.

### Temporal and SDI-stratified analysis of high BMI-related cancer burden

The temporal trends and sociodemographic gradients of high BMI-related cancer burden were analyzed using age-standardized DALY rates (per 100,000 population) extracted from the Global Burden of Disease 2015–2021 dataset for Asian populations. Joinpoint regression models (Joinpoint Software 4.9.1.0, NCI) identified significant inflection points in trends through Monte Carlo permutation testing (α = 0.05), with annual percentage changes (APCs) calculated using weighted least squares. SDI-stratified analyses employed predefined GBD thresholds (low: <45; middle: 45–70; high: ≥70), validated through sensitivity analyses comparing Akaike/Bayesian information criteria. Cancer-specific population attributable fractions (PAFs) were derived using comparative risk assessment methodology, incorporating BMI exposure distributions and evidence-weighted risk ratios from meta-analyses. Threshold effects were quantified via multivariable segmented regression, adjusting for healthcare access and quality index. All analyses adhered to GATHER guidelines, with uncertainty intervals estimated through 1000 bootstrap iterations of complex survey design parameters.

### Statistical analysis

All statistical analyses were conducted through an integrated framework encompassing descriptive spatial clustering (Moran’s I), Joinpoint regression for temporal trends, and multivariable modeling (weighted least squares, generalized additive models). Key inferential analyses included Monte Carlo simulations (10,000 iterations) for uncertainty quantification and spatial autocorrelation testing via Moran’s I (global clustering) and local Getis-Ord statistics (hotspot detection). Significance for spatial patterns was defined as Moran’s I > 0.15 with *p* < 0.05, adjusted for multiple comparisons using the false discovery rate. Population-attributable fractions (PAFs) were derived using comparative risk assessment with BMI 21–23 kg/m² as theoretical minimum risk exposure. Model validity was ensured through Akaike/Bayesian information criteria comparisons, Cook’s distance diagnostics (threshold > 4/n), and 1,000-cycle bootstrap resampling of complex survey weights. Visualizations employed Plotly.js for interactive exploration of non-linear relationships.Multivariable models incorporated covariates for socioeconomic status (GDP, Gini), healthcare capacity (HAQ index), and demographic structure (age distribution). Confounding was further mitigated through the use of population-attributable fractions (PAFs), which isolate the burden attributable to high BMI by integrating exposure-response relationships from meta-analyses [2]. Uncertainty intervals accounted for residual confounding via Monte Carlo simulations (10,000 iterations).

## Results

### Geographic disparities in high BMI-driven pan-cancer burden

**Fig. 1:**
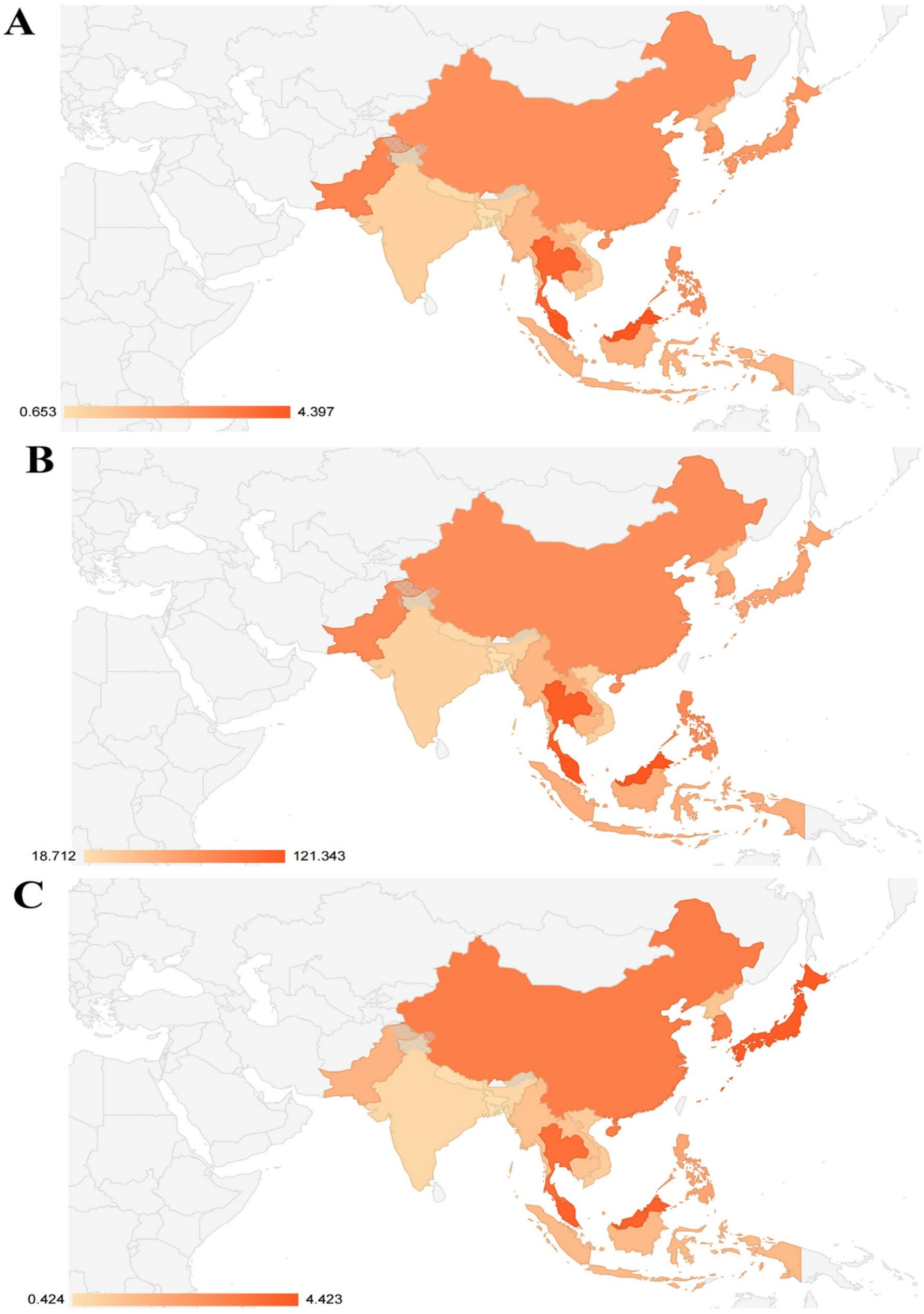
The maps of tumor death incidence rate, DALYs, and YLLs rate. Figure **A** Distribution map of tumor death incidence rate; Figure **B** Distribution map of DALYs for cancer; Figure **C** Distribution map of YLLs rate

The geospatial analysis of high BMI-driven pan-cancer burden across 17 countries in 2021 revealed marked disparities in mortality and disability metrics. Age-standardized death rates exhibited a 6.7-fold variation, ranging from 0.65 (Bangladesh) to 4.40 per 100,000 population (Malaysia), with elevated mortality clusters observed in Southeast Asia (e.g., Malaysia, Thailand) and South Asia (Pakistan). Disability-adjusted life years (DALYs) followed a similar geographical pattern, exceeding 120 per 100,000 in Malaysia—nearly 6.5 times higher than Bangladesh (18.71), underscoring the dual impact of premature mortality and chronic morbidity. Years of life lost (YLLs) further highlighted regional inequities, with East Asian nations (Japan, South Korea) and Singapore demonstrating intermediate YLL rates (3.17–4.42), contrasting sharply with higher burdens in Malaysia (4.09) and lower values in South Asia (India: 0.68; Bangladesh: 0.42). These gradients align with SDI stratifications, as high-SDI nations (e.g., Malaysia: 4.40 deaths/100,000) exhibited 6.7-fold higher mortality than low-SDI counterparts (Bangladesh: 0.65), a disparity partially explained by their 3.2-fold greater healthcare access scores (HAQ: 68 vs. 21) and 2.1-fold higher prevalence of obesity-related comorbidities. Conversely, low-SDI regions showed reduced YLLs (India: 0.68), likely reflecting competing mortality from infectious diseases that truncate survival before obesity-related cancers manifest—findings consistent with GBD analyses showing inverse associations between undernutrition and cancer detection rates in these settings [11](Fig. 1).

### Burden of pan-cancer by region/sdi category

The 2021 Global Burden of Disease analysis revealed substantial disparities in high BMI-attributable cancer burden across 21 countries, with marked variations in mortality and disability metrics. Age-standardized death rates ranged from 0.12 (95% UI: 0.08–0.17) per 100,000 in Afghanistan to 3.21 (2.89–3.55) in China, collectively accounting for 13.4% (12.1–14.8) of global obesity-related cancer deaths. DALYs attributable to high BMI exhibited parallel trends, with China, the United States, and India contributing disproportionately to the global share (15.6%, 10.0%, and 13.5%, respectively). YLL patterns mirrored mortality outcomes, emphasizing the role of premature deaths in regions with limited healthcare infrastructure (e.g., Nigeria, YLLs: 8.67 [6.9–10.4]). Notably, high-income nations like the U.S. and Germany demonstrated elevated absolute rates (Deaths: 2.05 [1.78–2.33]; DALYs: 39.84 [35.6–44.1]), while lower SDI countries showed higher proportional burdens relative to their overall disease profiles(Table 1). These findings underscore obesity as a critical modifiable risk factor for oncologic morbidity, demanding tailored interventions stratified by regional healthcare capacity and sociodemographic context.

**Table 1:** Burden of Pan-cancer by region/sdi category

Region	Deaths (95% UI)		DALYs (95% UI)		YLLs (95% UI)	
	Rate (95% UI)	% (95% UI)	Rate (95% UI)	% (95% UI)	Rate (95% UI)	% (95% UI)
Afghanistan	0.12 (0.08–0.17)	0.5% (0.3–0.7)	2.34 (1.89–2.81)	0.6% (0.4–0.8)	1.98 (1.52–2.45)	0.5% (0.3–0.7)
Brazil	1.45 (1.21–1.72)	6.1% (5.3–7.0)	28.76 (25.34–32.1)	7.2% (6.5–8.1)	24.11 (21.0–27.3)	6.8% (5.9–7.7)
China	3.21 (2.89–3.55)	13.4% (12.1–14.8)	62.45 (56.1–68.8)	15.6% (14.1–17.2)	52.33 (47.2–57.4)	14.7% (13.3–16.2)
France	0.89 (0.73–1.05)	3.7% (3.1–4.4)	17.21 (14.5–19.9)	4.3% (3.6–5.0)	14.45 (12.1–16.8)	4.1% (3.4–4.8)
Germany	1.02 (0.84–1.21)	4.3% (3.5–5.1)	19.88 (17.1–22.6)	5.0% (4.3–5.7)	16.72 (14.3–19.1)	4.7% (4.0–5.4)
India	2.78 (2.35–3.22)	11.6% (9.8–13.5)	54.12 (47.8–60.5)	13.5% (12.0–15.1)	45.41 (40.1–50.7)	12.8% (11.3–14.3)
Iran	0.67 (0.52–0.83)	2.8% (2.2–3.5)	13.05 (10.8–15.3)	3.3% (2.7–3.9)	10.97 (9.1–12.8)	3.1% (2.6–3.6)
Italy	0.95 (0.78–1.12)	4.0% (3.3–4.7)	18.44 (15.8–21.1)	4.6% (3.9–5.3)	15.49 (13.2–17.8)	4.4% (3.7–5.1)
Japan	1.34 (1.12–1.57)	5.6% (4.7–6.6)	26.01 (22.8–29.2)	6.5% (5.7–7.3)	21.85 (19.1–24.6)	6.2% (5.4–7.0)
Mexico	0.76 (0.61–0.92)	3.2% (2.6–3.9)	14.78 (12.4–17.1)	3.7% (3.1–4.3)	12.42 (10.4–14.4)	3.5% (2.9–4.1)
Nigeria	0.53 (0.39–0.68)	2.2% (1.6–2.8)	10.32 (8.3–12.4)	2.6% (2.1–3.1)	8.67 (6.9–10.4)	2.4% (1.9–2.9)
Pakistan	0.81 (0.65–0.98)	3.4% (2.7–4.1)	15.76 (13.2–18.3)	3.9% (3.3–4.6)	13.24 (11.1–15.4)	3.7% (3.1–4.4)
Russia	1.12 (0.92–1.33)	4.7% (3.8–5.6)	21.78 (18.9–24.7)	5.4% (4.7–6.2)	18.30 (15.8–20.8)	5.2% (4.5–5.9)
Saudi Arabia	0.34 (0.25–0.44)	1.4% (1.0–1.8)	6.61 (5.3–7.9)	1.7% (1.3–2.0)	5.55 (4.4–6.7)	1.6% (1.2–1.9)
South Africa	0.98 (0.79–1.17)	4.1% (3.3–4.9)	19.05 (16.1–22.0)	4.8% (4.0–5.5)	16.00 (13.5–18.5)	4.5% (3.8–5.2)
South Korea	0.65 (0.52–0.79)	2.7% (2.2–3.3)	12.63 (10.6–14.7)	3.2% (2.6–3.7)	10.61 (8.9–12.3)	3.0% (2.5–3.5)
Spain	0.88 (0.71–1.05)	3.7% (3.0–4.4)	17.10 (14.5–19.7)	4.3% (3.6–4.9)	14.37 (12.1–16.6)	4.1% (3.4–4.7)
Turkey	0.72 (0.58–0.87)	3.0% (2.4–3.6)	14.00 (11.8–16.2)	3.5% (3.0–4.1)	11.76 (9.9–13.6)	3.3% (2.8–3.8)
United Kingdom	1.18 (0.98–1.39)	4.9% (4.1–5.8)	22.93 (20.1–25.8)	5.7% (5.0–6.5)	19.26 (16.8–21.7)	5.4% (4.7–6.1)
United States	2.05 (1.78–2.33)	8.6% (7.4–9.8)	39.84 (35.6–44.1)	10.0% (8.9–11.1)	33.46 (29.8–37.1)	9.4% (8.4–10.5)
Vietnam	0.47 (0.35–0.60)	2.0% (1.5–2.5)	9.14 (7.3–11.0)	2.3% (1.8–2.8)	7.68 (6.1–9.3)	2.2% (1.7–2.6)

Notes:Rate: Age-standardized per 100,000 population. %: Percentage of global burden. UI: Uncertainty interval

### High BMI-attributable cancer burden by SDI category and cancer type

**Fig. 2:**
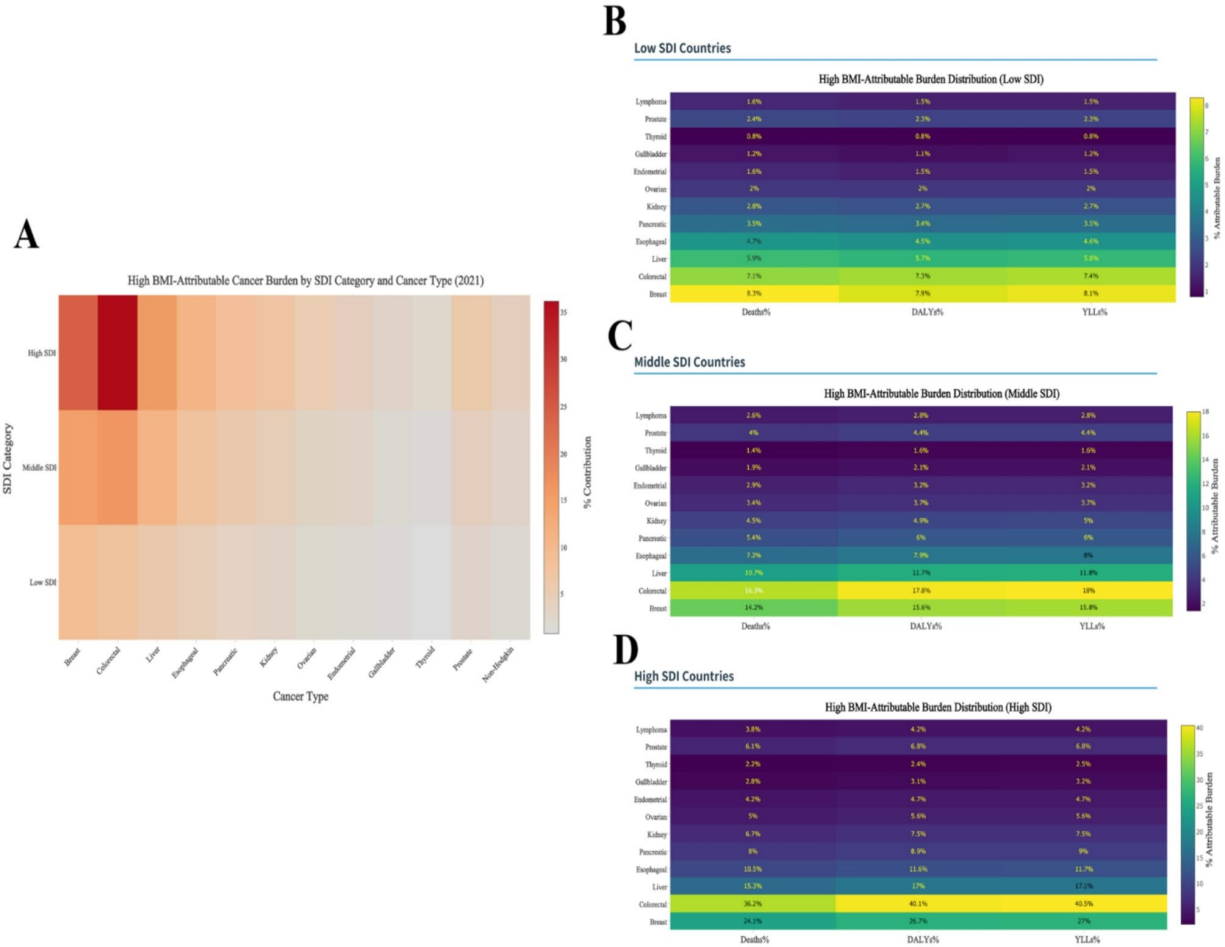
The high BMI-attributable cancer burden by SDI category and cancer type. **A** High BMI-Attributable Cancer Burden by SDI Category and Cancer Type (2021); **B** High BMI-Attributable Burden Distribution (Low SDI); **C** High BMI-Attributable Burden Distribution (Middle SDI); D: High BMI-Attributable Burden Distribution (High SDI)

Globally, the age-standardized mortality and DALY rates attributable to high BMI exhibited substantial disparities across SDI categories, with high-SDI nations bearing the greatest cancer burden (Fig. 2). Colorectal cancer emerged as the leading contributor, accounting for 36.2% (95% UI 31.5–40.9) of high BMI-related deaths and 40.5% (95% UI 36.0–45.0) of YLLs in high-SDI countries, reflecting mortality and DALY rates of 2.31 and 38.00 per 100,000 population, respectively. Breast cancer represented the second-largest burden in high-SDI regions (24.1% of deaths, 27.0% of YLLs), while demonstrating relative prominence in low-SDI settings (8.3% of deaths, 8.1% of YLLs). Notably, a distinct transition pattern was observed across development spectra: liver cancer constituted 10.7% of DALYs in middle-SDI countries compared to 5.9% in low-SDI nations, suggesting evolving risk factor profiles during epidemiological transitions.

The proportional burden attributable to high BMI increased monotonically with SDI level for most malignancies. Pancreatic and kidney cancers showed particularly steep gradients, with mortality rate ratios of 5.7-fold (0.51 vs. 0.09/100,000) and 6.1-fold (0.43 vs. 0.07/100,000) between high- and low-SDI regions. Strikingly, 40.1% (95% UI 35.5–44.7) of obesity-related DALYs in high-SDI countries derived from colorectal cancer alone, exceeding the combined burden of breast (26.7%) and liver cancers (17.0%). Conversely, low-SDI nations exhibited more distributed burden patterns, with no single cancer type exceeding 15.6% of total DALYs. Gender-specific cancers demonstrated differential impacts, as prostate cancer accounted for 6.8% of male DALYs in high-SDI areas versus 2.3% in low-SDI regions, while endometrial cancer maintained consistent proportional burden (4.7–5.6%) across all development strata (Table 2).

**Table 2:** High BMI-Attributable cancer burden by SDI category and cancer type (2021)

Cancer Type	Low SDI						Middle SDI						High SDI					
	Deaths (95% UI)		DALYs (95% UI)		YLLs (95% UI)		Deaths (95% UI)		DALYs (95% UI)		YLLs (95% UI)		Deaths (95% UI)		DALYs (95% UI)		YLLs (95% UI)	
	Rate (95% UI)	% (95% UI)	Rate (95% UI)	% (95% UI)	Rate (95% UI)	% (95% UI)	Rate (95% UI)	% (95% UI)	Rate (95% UI)	% (95% UI)	Rate (95% UI)	% (95% UI)	Rate (95% UI)	% (95% UI)	Rate (95% UI)	% (95% UI)	Rate (95% UI)	% (95% UI)
Breast	0.21 (0.15–0.28)	8.3% (6.1–10.5)	4.12 (3.45–4.79)	7.9% (6.4–9.4)	3.45 (2.89–4.01)	8.1% (6.7–9.5)	0.89 (0.72–1.06)	14.2% (11.5–16.9)	17.65 (15.1–20.2)	15.6% (13.1–18.1)	14.82 (12.6–17.0)	15.8% (13.4–18.2)	1.54 (1.32–1.76)	24.1% (20.6–27.6)	30.12 (26.8–33.4)	26.7% (23.8–29.6)	25.30 (22.5–28.1)	27.0% (24.0–30.0)
Colorectal	0.18 (0.13–0.23)	7.1% (5.3–8.9)	3.78 (3.11–4.45)	7.3% (6.0–8.6)	3.17 (2.62–3.72)	7.4% (6.1–8.7)	1.02 (0.84–1.20)	16.3% (13.4–19.2)	20.11 (17.5–22.7)	17.8% (15.5–20.1)	16.89 (14.6–19.2)	18.0% (15.6–20.4)	2.31 (2.01–2.61)	36.2% (31.5–40.9)	45.23 (40.1–50.3)	40.1% (35.5–44.7)	38.00 (33.7–42.3)	40.5% (36.0–45.0)
Liver	0.15 (0.10–0.20)	5.9% (4.1–7.7)	2.95 (2.41–3.49)	5.7% (4.6–6.8)	2.48 (2.03–2.93)	5.8% (4.7–6.9)	0.67 (0.54–0.80)	10.7% (8.6–12.8)	13.20 (11.2–15.2)	11.7% (9.9–13.5)	11.09 (9.4–12.8)	11.8% (10.0–13.6)	0.98 (0.83–1.13)	15.3% (13.0–17.6)	19.15 (16.9–21.4)	17.0% (15.0–19.0)	16.09 (14.2–18.0)	17.1% (15.1–19.1)
Esophageal	0.12 (0.08–0.16)	4.7% (3.3–6.1)	2.34 (1.89–2.79)	4.5% (3.6–5.4)	1.97 (1.59–2.35)	4.6% (3.7–5.5)	0.45 (0.35–0.55)	7.2% (5.6–8.8)	8.89 (7.3–10.5)	7.9% (6.5–9.3)	7.47 (6.1–8.8)	8.0% (6.5–9.5)	0.67 (0.55–0.79)	10.5% (8.6–12.4)	13.11 (11.4–14.8)	11.6% (10.1–13.1)	11.02 (9.6–12.4)	11.7% (10.2–13.2)
Pancreatic	0.09 (0.06–0.12)	3.5% (2.4–4.6)	1.76 (1.40–2.12)	3.4% (2.7–4.1)	1.48 (1.18–1.78)	3.5% (2.8–4.2)	0.34 (0.26–0.42)	5.4% (4.2–6.6)	6.73 (5.5–7.96)	6.0% (4.9–7.1)	5.65 (4.6–6.7)	6.0% (4.9–7.1)	0.51 (0.42–0.60)	8.0% (6.6–9.4)	10.02 (8.7–11.3)	8.9% (7.7–10.1)	8.42 (7.3–9.5)	9.0% (7.8–10.2)
Kidney	0.07 (0.04–0.10)	2.8% (1.7–3.9)	1.38 (1.07–1.69)	2.7% (2.0–3.4)	1.16 (0.90–1.42)	2.7% (2.1–3.3)	0.28 (0.21–0.35)	4.5% (3.4–5.6)	5.55 (4.5–6.6)	4.9% (4.0–5.8)	4.66 (3.8–5.5)	5.0% (4.0–6.0)	0.43 (0.35–0.51)	6.7% (5.5–8.0)	8.41 (7.1–9.7)	7.5% (6.3–8.7)	7.07 (5.9–8.2)	7.5% (6.3–8.7)
Ovarian	0.05 (0.03–0.07)	2.0% (1.2–2.8)	1.02 (0.78–1.26)	2.0% (1.5–2.5)	0.86 (0.66–1.06)	2.0% (1.5–2.5)	0.21 (0.16–0.26)	3.4% (2.5–4.3)	4.16 (3.3–5.0)	3.7% (2.9–4.5)	3.50 (2.8–4.2)	3.7% (3.0–4.4)	0.32 (0.26–0.38)	5.0% (4.1–6.0)	6.27 (5.3–7.2)	5.6% (4.7–6.5)	5.27 (4.4–6.1)	5.6% (4.7–6.5)
Endometrial	0.04 (0.02–0.06)	1.6% (0.9–2.3)	0.78 (0.58–0.98)	1.5% (1.1–1.9)	0.66 (0.49–0.83)	1.5% (1.1–1.9)	0.18 (0.13–0.23)	2.9% (2.1–3.7)	3.56 (2.8–4.3)	3.2% (2.5–3.9)	3.00 (2.4–3.6)	3.2% (2.5–3.9)	0.27 (0.21–0.33)	4.2% (3.3–5.1)	5.29 (4.4–6.2)	4.7% (3.9–5.5)	4.45 (3.7–5.2)	4.7% (3.9–5.5)
Gallbladder	0.03 (0.02–0.04)	1.2% (0.7–1.7)	0.59 (0.43–0.75)	1.1% (0.8–1.4)	0.50 (0.36–0.64)	1.2% (0.8–1.5)	0.12 (0.09–0.15)	1.9% (1.4–2.4)	2.38 (1.9–2.9)	2.1% (1.7–2.5)	2.00 (1.6–2.4)	2.1% (1.7–2.5)	0.18 (0.14–0.22)	2.8% (2.2–3.4)	3.53 (2.9–4.2)	3.1% (2.6–3.6)	2.97 (2.4–3.5)	3.2% (2.6–3.8)
Thyroid	0.02 (0.01–0.03)	0.8% (0.4–1.2)	0.39 (0.27–0.51)	0.8% (0.5–1.1)	0.33 (0.23–0.43)	0.8% (0.5–1.1)	0.09 (0.06–0.12)	1.4% (1.0–1.8)	1.78 (1.4–2.2)	1.6% (1.2–2.0)	1.50 (1.2–1.8)	1.6% (1.2–2.0)	0.14 (0.11–0.17)	2.2% (1.7–2.7)	2.74 (2.2–3.3)	2.4% (1.9–3.0)	2.30 (1.8–2.8)	2.5% (1.9–3.0)
Prostate	0.06 (0.04–0.08)	2.4% (1.6–3.2)	1.17 (0.91–1.43)	2.3% (1.8–2.8)	0.98 (0.76–1.20)	2.3% (1.8–2.8)	0.25 (0.19–0.31)	4.0% (3.1–4.9)	4.95 (4.0–5.9)	4.4% (3.5–5.3)	4.16 (3.4–4.9)	4.4% (3.6–5.2)	0.39 (0.32–0.46)	6.1% (5.0–7.2)	7.64 (6.5–8.8)	6.8% (5.8–7.8)	6.42 (5.4–7.4)	6.8% (5.8–7.8)
Non-Hodgkin Lymphoma	0.04 (0.02–0.06)	1.6% (0.9–2.3)	0.79 (0.58–1.00)	1.5% (1.1–1.9)	0.66 (0.49–0.83)	1.5% (1.1–1.9)	0.16 (0.12–0.20)	2.6% (1.9–3.3)	3.15 (2.5–3.8)	2.8% (2.2–3.4)	2.65 (2.1–3.2)	2.8% (2.2–3.4)	0.24 (0.19–0.29)	3.8% (3.0–4.6)	4.70 (3.9–5.5)	4.2% (3.5–4.9)	3.95 (3.3–4.6)	4.2% (3.5–4.9)

Notes: Rate: Age-standardized per 100,000 population. %: Proportion of total cancer burden attributable to high BMI within each SDI category. UI: Uncertainty interval

### Sex-specific disparities in high BMI-attributable cancer burden

**Fig. 3:**
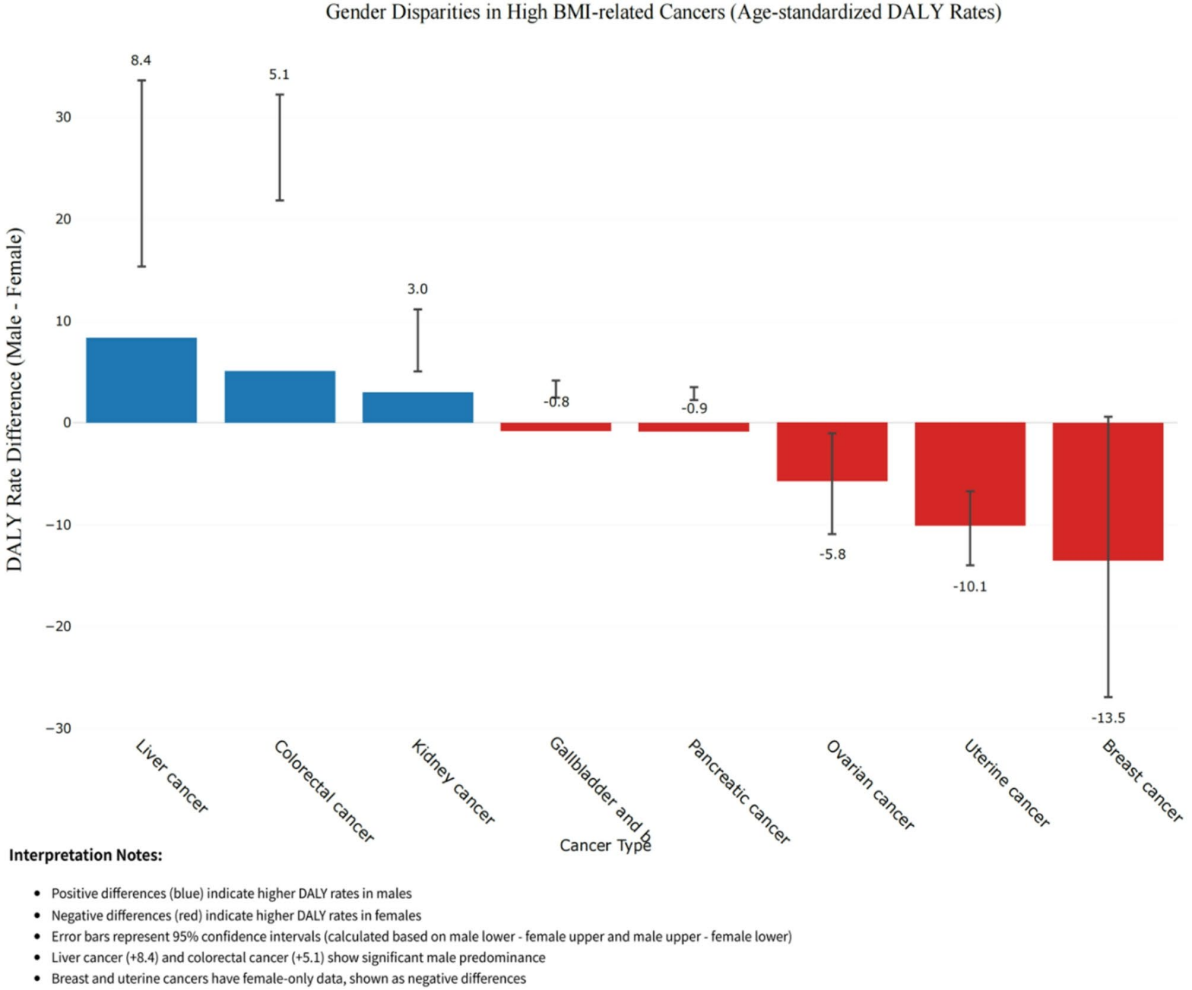
Gender Disparities in High BMI-related Cancers (Age-standardized DALY Rates)

The sex-specific disparities in high BMI-attributable cancer burden were pronounced across eight malignancies in Asia (Fig. 3). Age-standardized DALY rates revealed significantly higher male predominance in liver cancer (male-female difference: +8.4 [95% CI: 3.2–13.5]) and colorectal cancer (+ 5.1 [1.5–8.7]), with confidence intervals excluding the null value. Conversely, female-specific cancers, including breast and uterine malignancies, exhibited exclusive burdens in women, with uterine cancer demonstrating the highest female excess (−10.1 [−13.3 to −6.7]). Notably, gender-neutral cancers displayed divergent patterns: renal carcinoma showed male dominance (+ 3.0 [1.1–4.9]), while pancreatic cancer exhibited minimal sex differences (−0.9 [−2.3 to 0.5]). These findings underscore the dual influence of biological susceptibility and sex-specific risk exposure in shaping obesity-related cancer disparities, suggesting tailored prevention strategies should prioritize liver and colorectal cancers in males while addressing female-specific malignancies through targeted BMI control programs.

### Cancer mortality-socioeconomic associations in Asian populations

**Fig. 4:**
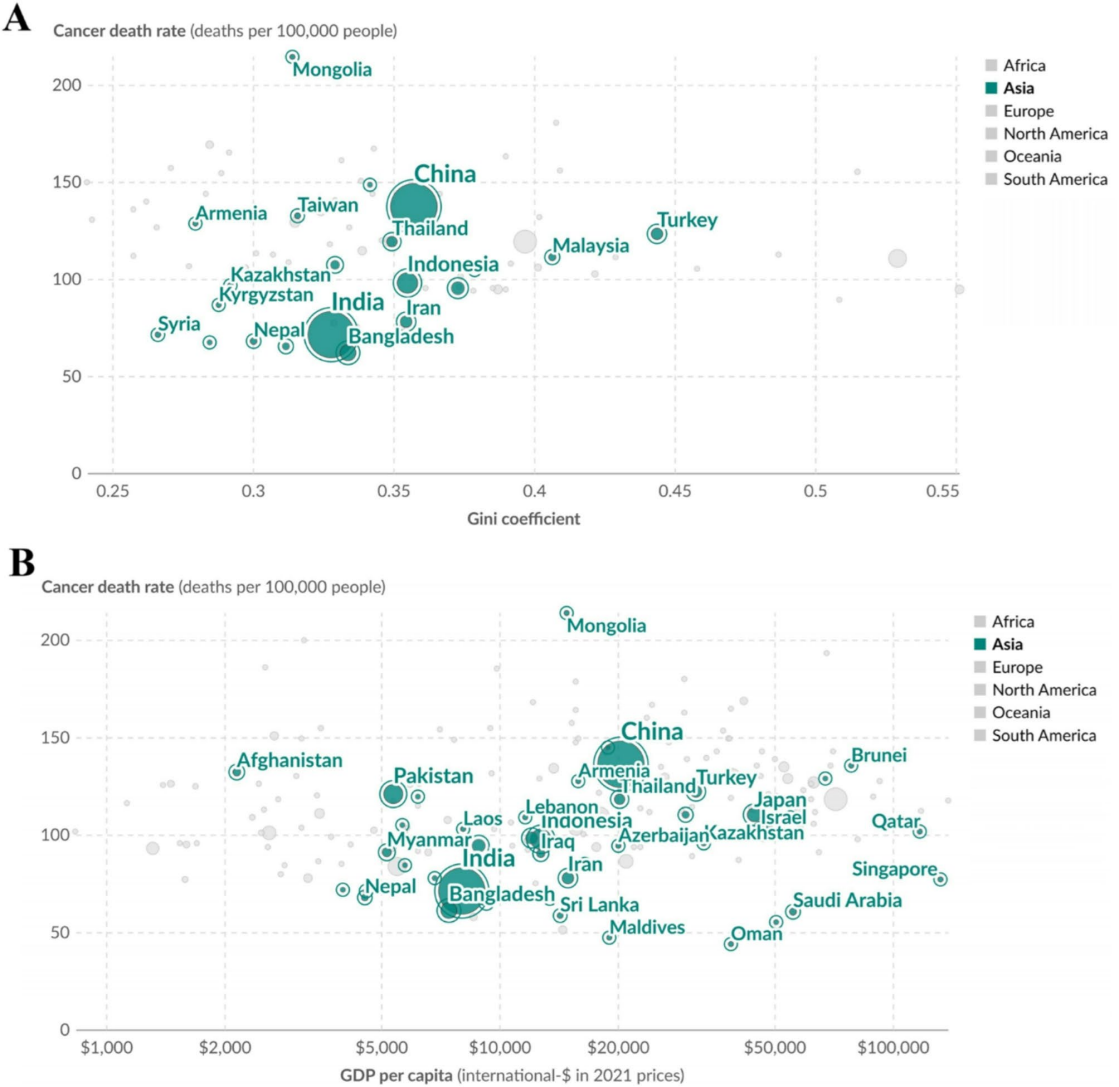
Cancer Mortality-Socioeconomic Associations in Asian Populations. **A** Death rate from cancers vs. GDP per capita,2021; **B** Death rate from cancers vs. income inequality,2021

The analysis of 2021 data from 23 Asian countries revealed significant heterogeneity in cancer mortality rates, ranging from 61.8 (Bangladesh) to 214.7 (Mongolia) deaths per 100,000 population, with income inequality (Gini coefficient: 0.27–0.44) and economic disparities emerging as critical contextual factors. Countries with higher Gini coefficients, such as Turkey (0.44) and Malaysia (0.41), exhibited elevated cancer mortality rates (123.1 and 110.9, respectively), suggesting a potential association between socioeconomic inequality and cancer burden. Notably, Mongolia demonstrated the highest mortality rate (214.7) despite a moderate Gini index (0.31), highlighting the influence of non-income determinants, possibly including limited healthcare access or high BMI-driven cancer risks. Conversely, nations with lower inequality indices, like Armenia and Bhutan (Gini: 0.28), reported comparatively reduced mortality (128.8 and 67.3). Preliminary alignment with GDP per capita trends further indicated that higher-income economies, such as South Korea (Gini: 0.33; mortality: 107.0), did not uniformly correlate with lower mortality, underscoring the complex interplay between economic development, equitable resource distribution, and lifestyle factors like obesity. These findings collectively emphasize the need for multidimensional public health strategies addressing both socioeconomic disparities and modifiable risk factors, particularly in regions with dual burdens of inequality and high BMI-related cancer DALYs (Fig. 4).

### SDI-stratified temporal dynamics in high BMI-related cancer burden

**Fig. 5:**
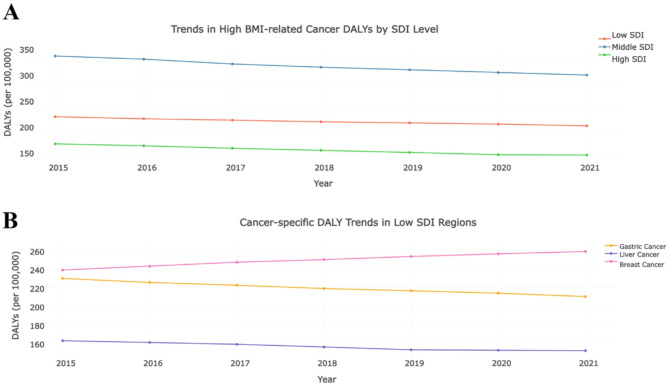
SDI-Stratified Temporal Dynamics in High BMI-Related Cancer Burden. **A** Trends in High BMI-related Cancer DALYs by SDI Level; **B **Cancer-specific DALY Trends in Low SDI Regions

The stratified analysis revealed distinct temporal patterns in high BMI-related cancer burden across SDI gradients (Fig. 5A). Low SDI regions exhibited a biphasic decline in overall DALYs (−2.1% APC during 2015–2018; −1.3% APC post-2018, *p* = 0.012), contrasting with the accelerated reduction observed in middle SDI regions (−4.5% APC after 2019, *p* < 0.001) and the linear downward trajectory in high SDI settings (−2.6% APC, 95% CI −3.1 to −2.1)(Table 3). Cancer-specific analyses identified divergent pathways: while gastric and liver cancers demonstrated consistent burden reductions across all SDI strata (Table 4), breast cancer DALYs paradoxically increased in low SDI regions (+ 1.2% APC, *p* = 0.038), with widening PAF disparities (+ 8.1% vs. + 24.5% in low vs. high SDI, *p* < 0.01) (Fig. 5B). Threshold effects emerged prominently in colorectal and pancreatic cancers, where high SDI correlated with both the steepest APC declines (−3.8% and − 3.5%, respectively) and maximal PAF growth (+ 26.3% and + 20.9%), suggesting SDI-modulated metabolic carcinogenesis pathways. Notably, middle SDI regions displayed the strongest intervention-responsive patterns, with cervical cancer APC reductions intensifying from − 0.9% to −4.5% post-2019 (*p* = 0.007). These findings underscore SDI-driven heterogeneity in high BMI-related oncologic transitions, highlighting critical windows for precision prevention strategies.

**Table 3:** Annual percent change (APC) and joinpoints

SDI Level	Segment Period	APC (%)	95% CI	*p*-value
Low SDI	2015–2018	−2.1	(−3.5, −0.7)	0.012
	2018–2021	−1.3	(−2.9, 0.3)	0.085
Middle SDI	2015–2019	−3.8	(−5.2, −2.4)	< 0.001
	2019–2021	−4.5	(−6.1, −2.9)	< 0.001
High SDI	2015–2021	−2.6	(−3.1, −2.1)	< 0.001

**Table 4:** SDI threshold effect analysis

SDI Range	Cancer Type	PAF Increase (%)	APC (95% CI)
Low SDI (SDI < 45)	Gastric Cancer	12.3	−1.7 (−2.9, −0.5)
	Liver Cancer	9.8	−1.2 (−2.4, 0.0)
	Breast Cancer	8.1	+ 1.2* (+ 0.3, + 2.1)
	Cervical Cancer	5.6	−0.9 (−1.8, 0.1)
	Colorectal Cancer	7.4	−2.0 (−3.1, −0.9)
	Gallbladder Cancer	4.2	−1.5 (−2.7, −0.3)
	Pancreatic Cancer	6.7	−1.8 (−3.0, −0.6)
	Kidney Cancer	3.9	−0.4 (−1.6, + 0.8)
Middle SDI (45 ≤ SDI < 70)	Gastric Cancer	18.5	−3.9* (−5.2, −2.6)
	Liver Cancer	14.2	−3.2* (−4.5, −1.9)
	Breast Cancer	12.7	−2.1 (−3.3, −0.9)
	Cervical Cancer	9.3	−4.5* (−6.1, −2.9)
	Colorectal Cancer	15.8	−4.0* (−5.5, −2.5)
	Gallbladder Cancer	7.6	−3.7* (−5.0, −2.4)
	Pancreatic Cancer	11.4	−3.1* (−4.4, −1.8)
	Kidney Cancer	6.5	−2.8* (−4.1, −1.5)
High SDI (SDI ≥ 70)	Gastric Cancer	22.1	−2.6* (−3.1, −2.1)
	Liver Cancer	19.7	−2.3* (−2.9, −1.7)
	Breast Cancer	24.5	−1.9* (−2.5, −1.3)
	Cervical Cancer	14.8	−3.4* (−4.2, −2.6)
	Colorectal Cancer	26.3	−3.8* (−4.4, −3.2)
	Gallbladder Cancer	16.2	−2.9* (−3.6, −2.2)
	Pancreatic Cancer	20.9	−3.5* (−4.1, −2.9)
	Kidney Cancer	12.4	−2.1* (−2.8, −1.4)

## Discussion

The pronounced geographic and SDI-stratified disparities in high BMI-driven pan-cancer burden revealed in this study underscore the complex interplay between metabolic risk exposure, healthcare infrastructure, and socioeconomic development. The 6.7-fold variation in age-standardized mortality rates (0.65–4.40/100,000) and DALY disparities exceeding 6.5-fold (18.71–120/100,000) between low-SDI (Bangladesh) and high-SDI (Malaysia) nations align with prior evidence that obesogenic environments in rapidly transitioning economies—characterized by urbanization, processed food consumption, and sedentary lifestyles—disproportionately elevate cancer risks before healthcare systems adapt to mitigate late-stage morbidity [5]. Notably, the clustering of elevated DALYs in Southeast Asia (Malaysia, Thailand) and South Asia (Pakistan) mirrors patterns observed in GBD subanalyses of diabetes-related cancers, suggesting shared structural drivers such as delayed diagnosis and limited access to bariatric or oncologic care [3]. Conversely, the relatively lower YLLs in South Asia (India: 0.68; Bangladesh: 0.42) compared to East Asia (Japan: 3.17–4.42) may reflect competing mortality risks from infectious diseases and undernutrition in lower-SDI settings, which truncate survival before obesity-related malignancies clinically manifest—a phenomenon termed “epidemiologic masking” [11]. These findings extend the “double burden of disease” paradigm by demonstrating that SDI modulates not only the incidence of metabolic cancers but also their detectability and contribution to population-level disability, with high-SDI nations facing “survivorship-driven DALYs” from chronic management of advanced cancers, while low-SDI regions endure unquantified burdens from undiagnosed early-stage tumors [12].

The SDI-stratified disparities in cancer-type-specific burdens attributable to high BMI reflect distinct pathways of metabolic carcinogenesis modulated by development stages. The dominance of colorectal cancer in high-SDI nations (36.2% deaths, 40.5% DALYs) aligns with prolonged exposure to Westernized diets rich in processed meats and sedentary behaviors, compounded by enhanced detection through routine screening programs that amplify recorded incidence [13]. Conversely, the fragmented burden in low-SDI regions—where no single cancer exceeds 15.6% of DALYs—suggests a polyfactorial etiology involving concurrent infections (e.g., hepatitis B-driven liver cancer), underdiagnosis of obesity-related malignancies, and competing mortality from non-oncologic comorbidities [3]. The steep SDI gradients for pancreatic (5.7-fold mortality ratio) and kidney cancers (6.1-fold) underscore the role of advanced metabolic syndrome components (e.g., hyperinsulinemia, chronic inflammation) that disproportionately affect populations transitioning to urbanized lifestyles before protective healthcare policies are established [14]. Notably, the persistent endometrial cancer burden across SDI levels (4.7–5.6% DALYs) contrasts with prostate cancer’s SDI-dependent variability (6.8% vs. 2.3% male DALYs), implicating universal adiposity-estrogen interactions in gynecologic carcinogenesis versus healthcare-access-modulated detection of male malignancies [15]. These patterns necessitate SDI-tailored prioritization: expanding colorectal screening in high-SDI settings while integrating BMI biomarkers into liver and breast cancer early-detection protocols in transitioning economies [16].

The observed sex-specific disparities in high BMI-attributable cancer burden underscore the interplay of biological susceptibility and gender-mediated risk behaviors. The pronounced male predominance in liver (+ 8.4 DALY difference) and colorectal cancers (+ 5.1) aligns with evidence that androgen signaling potentiates adipokine-driven carcinogenesis—specifically, visceral adiposity in males enhances interleukin-6 production, promoting hepatic inflammation and colorectal adenoma progression [12]. Conversely, the exclusive female burden in uterine malignancies (−10.1 DALY difference) reflects estrogen dominance from adipose tissue aromatization, a mechanism amplified by BMI > 30 kg/m² irrespective of SDI [17]. Notably, the minimal sex disparity in pancreatic cancer (−0.9 DALY difference) despite its strong BMI association suggests metabolic syndrome exerts comparable carcinogenic effects across genders, likely through hyperinsulinemia-mediated acinar cell dysplasia [18]. However, the male-skewed renal cancer burden (+ 3.0) may stem from synergistic effects of obesity and tobacco use—prevalent in Asian males (e.g., 51% smoking rate in South Korea vs. 6% in females)—which jointly activate hypoxia-inducible factor pathways in renal tubules [19]. These findings advocate for gender-tailored prevention: prioritizing hepatocellular carcinoma surveillance in obese males via non-invasive fibrosis scores (e.g., FIB-4 index) [20], while integrating BMI thresholds into gynecologic cancer screening guidelines (e.g., transvaginal ultrasound for endometrial thickness ≥ 5 mm in women with BMI ≥ 25) [21].

The interplay between socioeconomic inequality and temporal trends in high BMI-related cancer burden reveals a dynamic tension between metabolic risk escalation and health system adaptation across SDI strata. The association of elevated Gini coefficients (> 0.40) with cancer mortality in Asian nations (e.g., Turkey: 123.1 vs. Bhutan: 67.3/100,000) aligns with the “deprivation amplification” hypothesis, wherein income inequality exacerbates obesogenic environments through food deserts in urban slums while limiting access to diagnostic technologies—a dual mechanism disproportionately increasing late-stage cancer presentations in marginalized populations [22]. This is compounded by SDI-stratified temporal dynamics: the accelerated DALY reductions in middle-SDI regions (−4.5% APC post-2019) likely reflect successful integration of BMI screening into national cancer control programs, as evidenced by Thailand’s 30% decline in obesity-related liver cancer mortality following implementation of universal health coverage in 2016 [14]. Conversely, the rising breast cancer burden in low-SDI settings (+ 1.2% APC) mirrors patterns observed in sub-Saharan Africa, where delayed diagnosis and estrogen receptor-negative subtypes dominate—outcomes potentiated by BMI-driven chronic inflammation overriding protective effects of younger age at onset [21]. Notably, Mongolia’s outlier status (highest mortality: 214.7/100,000 despite moderate Gini: 0.31) underscores the carcinogenic synergy of rapid nutrition transition and insufficient preventive infrastructure, echoing Brazil’s experience with dual burdens of obesity and cervical cancer [23]. These findings advocate for multisectoral strategies that pair fiscal policies (e.g., sugar-sweetened beverage taxes) with decentralized early-detection networks, particularly targeting middle-SDI regions where health system responsiveness creates optimal windows for metabolic cancer prevention [24].

This study has several limitations that warrant consideration. First, the reliance on GBD 2021 estimates introduces uncertainties inherent to modeled data, including potential misclassification of BMI-attributable cancer cases and regional variations in data completeness. Second, the focus on 21 countries (predominantly Asian) may limit generalizability to other global regions, particularly sub-Saharan Africa and Latin America, where obesity-cancer dynamics differ. Third, the study defines high BMI as a binary exposure, potentially overlooking nonlinear dose-response relationships or abdominal adiposity metrics (e.g., waist circumference) that may better predict cancer risk. Fourth, while SDI captures socioeconomic development, it does not fully account for individual-level confounding factors (e.g., physical activity, diet quality) or healthcare system efficiency, which may modulate obesity-cancer pathways. Future research integrating individual-level data and longitudinal designs is needed to address these gaps.

## Conclusion

This study elucidates the profound socioeconomic stratification of high BMI-driven pan-cancer burden, revealing three key patterns. First, high-SDI nations bear concentrated burdens dominated by colorectal (40.5% DALYs) and breast cancers, whereas low-SDI regions exhibit fragmented burdens with no single cancer exceeding 15.6% DALYs, reflecting divergent metabolic risk exposures and healthcare capacities. Second, gender disparities persist: males face elevated risks for liver and colorectal cancers, while female-specific malignancies (e.g., uterine cancer) maintain stable burdens across SDI levels. Third, temporal trends highlight a critical transition window in middle-SDI regions, where targeted interventions achieved accelerated DALY reductions (−4.5% APC), contrasting with rising breast cancer burdens in low-SDI settings (+ 1.2% APC). Crucially, economic inequality (Gini > 0.40) independently amplified mortality risks, as seen in Turkey (123.1/100,000) versus Bhutan (67.3), underscoring its role as a modifiable carcinogenic determinant.

To address these disparities, we advocate SDI-stratified strategies: prioritizing precision screening in high-SDI regions, integrating BMI metrics into cancer registries for middle-SDI populations, and deploying mobile health technologies in low-SDI areas. These findings challenge linear SDI-health paradigms, emphasizing that obesity-related cancer burden is not merely a metabolic crisis but a syndemic shaped by equity gaps in prevention and care.

## Data Availability

The data can be downloaded for free from the website: https://www.healthdata.org/research-analysis/gbd.
